# Comparison of Medial Temporal Measures between Binswanger's Disease and Alzheimer's Disease

**DOI:** 10.1371/journal.pone.0086423

**Published:** 2014-01-23

**Authors:** Xuntao Yin, Chen Liu, Li Gui, Lu Zhao, Jiuquan Zhang, Luqing Wei, Bing Xie, Daiquan Zhou, Chuanming Li, Jian Wang

**Affiliations:** 1 Department of Radiology, Southwest Hospital, Third Military Medical University, Chongqing, China; 2 McConnell Brain Imaging Centre, Montreal Neurological Institute, McGill University, Montreal, Quebec, Canada; 3 Department of Neurology, Southwest Hospital, Third Military Medical University, Chongqing, China; University of Manchester, United Kingdom

## Abstract

Binswanger's disease (BD) is a common cause of vascular dementia in elderly patients; however, few studies have investigated the medial temporal lobe (MTL) atrophy in BD, and the differences in the atrophic patterns between BD and Alzheimer's disease (AD) remain largely unknown. Such knowledge is essential for understanding the pathologic basis of dementia. In this study, we collected structural magnetic resonance imaging (MRI) data from 16 normal controls, 14 patients with AD and 14 patients with BD. The volumes of the hippocampus and amygdala, and morphologic parameters (volume, surface area, cortical thickness and mean curvature) of the entorhinal cortex (ERC) and perirhinal cortex (PRC) were calculated using an automated approach. Volume reduction of the hippocampus, amygdala and ERC, and disturbance of the PRC curvature was found in both AD and BD patients compared with the controls (*p*<0.05, uncorrected). There were no significant differences among all the structural measures between the AD and BD patients. Finally, partial correlation analyses revealed that cognitive decline could be attributed to ERC thinning in AD and volume reduction of PRC in BD. We conclude that AD and BD exhibit similar atrophy patterns in the medial temporal cortices and deep gray matter but have distinct pathologic bases for cognitive impairments. Although atrophy of the MTL structures is a sensitive biomarker for AD, it is not superior for discrimination between AD and BD.

## Introduction

Vascular dementia (VaD) has become the second most common type of dementia after Alzheimer's disease (AD) and accounts for approximately 30% of dementia [1]. VaD with small vessel disease, also known as subcortical ischemic vascular dementia (SIVD), can be divided into two types, lacunar state and Binswanger's disease (BD, subcortical arteriosclerotic encephalopathy) [2]. The main vascular pathology of BD is lipo- or fibrohyalinosis in the medullary arteries, resulting in widespread infarction of deep white matter [3]. In contrast to the sudden development of dementia in the lacunar state, the slowly progressive dementia in BD may be mistaken for AD [4]. Therefore, elucidating the distinctively impaired patterns of brain structures in BD and AD will help to identify the pathological substrates of cognitive impairments. Such knowledge is also essential for determining the specificity of imaging markers for the diagnosis of dementia.

Atrophy of the medial temporal structures is now considered to be valid diagnostic criterion for AD. In particular, hippocampal atrophy is among the best established structural magnetic resonance imaging (MRI) markers [5]. However, increasing evidence suggests that hippocampal atrophy occurs in both SIVD and AD [6]–[8]. Hippocampal changes also occur commonly in other forms of cerebrovascular diseases that have a high risk of progression to VaD, such as cerebral small vessel disease [9], CADASIL [10] and brain infarcts [11]. Therefore, the hippocampus may also be the neurodegenerative site in BD. Besides, one pioneering study using voxel-based morphometry also found atrophy of the amygdala in both AD and BD patients [8]. However, direct comparisons between the two groups regarding the atrophic degrees in total volume of the hippocampus or amygdala have not been performed.

The entorhinal cortex (ERC) and perirhinal cortex (PRC), which connect the neocortex and the hippocampal formation [12], are also involved in the pathology of AD. From the histological perspective, the ERC is one of the earliest structures demonstrating neurofibrillary tangles in AD [13]. Neuroimaging studies also revealed that AD subjects had smaller ERC volume [14]–[17] or thickness [18], which likely contributed to memory impairment [19]. Furthermore, the decreased volume [20], [21] or inward surface deformation [22] of the ERC in patients with mild cognitive impairment (MCI) could contribute to the conversion to AD. In addition to the ERC, the PRC also manifests histological [23], volume [16], [17] and thickness [24] changes in AD. Nevertheless, in contrast to the well-documented ERC and PRC atrophy in AD, there is currently no consensus concerning whether these regions are involved in BD, mainly due to the pathologic diversity among the subtypes of VaD and the difficulties in the delineation of these structures on MR images.

Therefore, the aims of this study were to examine the structural impairment of BD in the cortices and deep gray matter of the medial temporal lobe (MTL), and to further compare the impaired patterns and severities between BD and AD. To avoid manual errors or protocol variations for the delineation of the medial temporal structures, we employed FreeSurfer for automatic segmentation. This method has been validated by histological examination [25], and could identify similar patterns of MTL atrophy in AD relative to manual segmentation [26], [27]. Therefore, it has been widely used to examine the MTL atrophy in AD [18], [21].

## Materials and Methods

### Ethics statement

All research procedures were approved by the Institutional Review Board of the Third Medical Military University and were conducted in accordance with the Declaration of Helsinki. The individuals in this manuscript have given written informed consent (as outlined in PLOS consent form) to participate in this study and publish these case details. Because cognitive disability can make it impossible to obtain valid informed consent, we also acquired written informed consent from the patients' surrogates (spouse or child). All potential participants who declined to participate or otherwise did not participate were eligible for treatment (if applicable) and were not disadvantaged in any other way by not participating in the study.

### Subjects

The patients with AD or BD were recruited from the Department of Neurology in Southwest Hospital, Chongqing between December 2010 and September 2012. Participants with evidence of severe psychiatric comorbidities, severe cognitive impairments (inability to perform neuropsychological tests) and use of medicines likely to affect cognitive function were excluded. Patients with probable AD fulfilled the National Institute of Neurological and Communicative Disorders and Stroke and the Alzheimer's Disease and Related Disorders Association (NINCDS-ADRDA) criteria [28]. Previous meta-analysis on pathologically verified dementia showed a high sensitivity and specificity when the cutoff of Hachinski Ischemic Score (HIS) was ≤4 for AD and ≥7 for VaD [29]. Therefore, AD patients who had HIS >4 (n = 5) were excluded from the study. We then excluded the patients with deep white matter legions and/or lacunar infarctions on MR images (n = 2).

BD patients were diagnosed according to the criteria proposed by Erkinjuntti et al [30]. All BD patients had clinical symptoms assessed by a series of neuropsychological examinations, and confluent white matter lesions (grade 2 or 3 according to the grading scale presented by Fazekas et al. [31]), which were identified by T2-weighted/fluid-attenuated inversion-recovery (FLAIR) MRI as areas of high signal intensity [32]. The subjects who had multiple lacunes (>5) in the deep gray matter were classified as lacunar state [30] and excluded from the study (n = 9). In order to exclude patients with “mixed” dementia, we also excluded the cases with a HIS <7 (n = 2) in the BD group.

The inclusion criteria for elderly control subjects included normal neurological examination, normal cognition as determined by neuropsychological tests, Mini Mental State Examination (MMSE) score ≥27 and Montreal Cognitive Assessment (MoCA) score ≥26 [33]. This study finally included 44 right-handed subjects, including 14 patients with AD, 14 patients with BD and 16 normal controls (Table 1).

**Table 1:** Demographic Data of the participants.

Characteristics	NC (n = 16)	AD (n = 14)	BD (n = 14)	*p* value
Gender (male/female)	10/6	9/5	9/5	0.92^a^
Age (years)	68.5±3.7	68.9±7.3	69.2±6.6	0.78^a^
Education (years)	9.2±2.8	8.2±3.4	6.8±5.4	0.26^a^
HRSD-17	6.8±2.2	8.2±2.5	7.7±1.7	0.2^a^
MMSE	28.3±1.2	18.1±4.4	15.5±3.9	0.11^b^
MoCA	27.4±1.5	12.5±6.2	9.1±3.8	0.09^b^
HIS	N/A	3.2±1.0	9.9±2.0	<0.001^b^
GDS	0	4.4±1.0	4.6±0.9	0.57^b^
CDR	0	1.6±0.9	1.6±0.6	0.95^b^
ADL/IADL	20	42.4±16.5	48.1±16.4	0.39^b^

Data were expressed as the mean ± standard deviation. ADL/IADL, Activity of Daily Living and Instrumental Activities of Daily Living scale; CDR, Clinical Dementia Rating; GDS, Global Deterioration Scale; HIS, Hachinski Ischemic Score; HRSD-17, Hamilton Rating Scale for Depression with17 items; MMSE, Mini Mental State Examination; MoCA, Montreal Cognitive Assessment; N/A, not applicable; NC, normal controls.

a The *p* value for ANOVA or chi-square test in 3 groups.

b The *p* value was obtained by two-sample t-test between the AD and BD patients.

### Image acquisition

Participants were scanned using a 3T scanner (MAGNETOM Trio Tim System, Siemens, Erlangen, Germany) with an 8-channel head coil. Magnetization-prepared rapid gradient echo (MPRAGE) sagittal images were collected by using the following parameters: repetition time  = 1, 900 ms, echo time  = 2.52 ms, inversion time  = 900 ms, flip angle  = 9°, matrix  = 256×256, thickness  = 1.0 mm, no gap, 176 slices, voxel size  = 1×1×1 mm^3^. FLAIR and T_2_-weighted images were also collected for inspection of anatomical abnormalities.

### Image analysis

**Figure 1:**
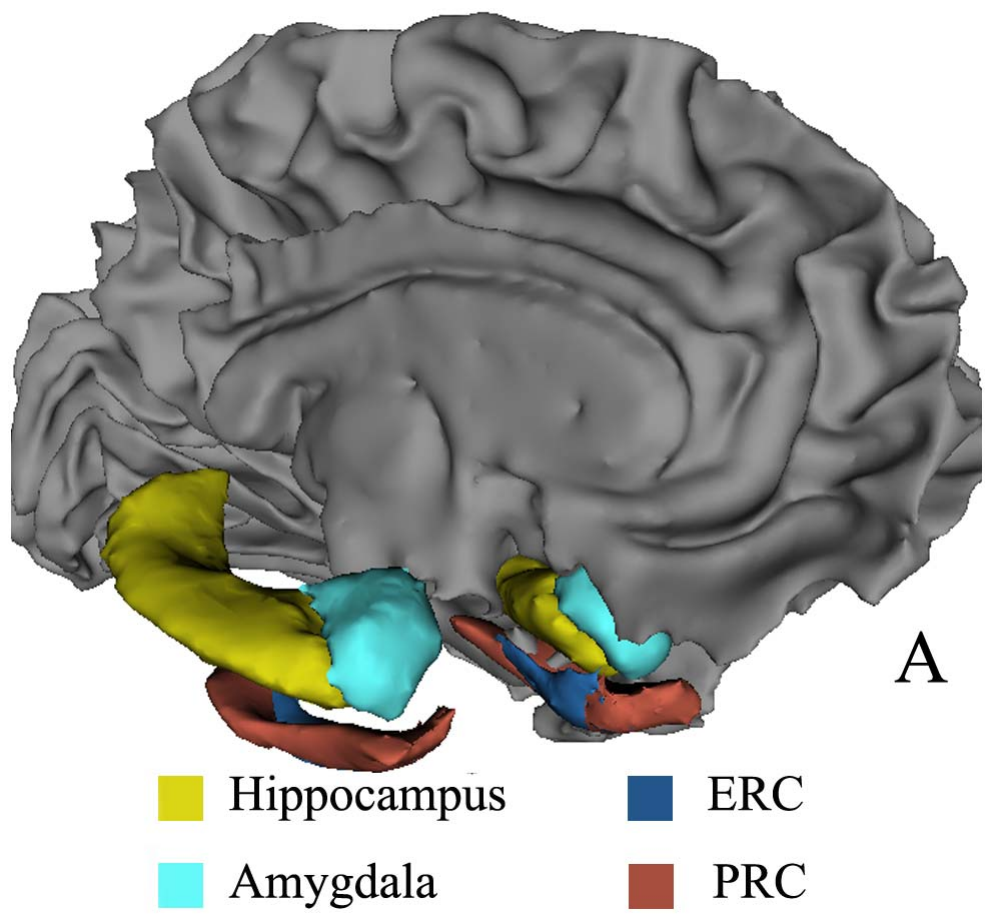
Medial temporal cortices and deep gray matter. These structures were overlapped on the left white matter surface. A, anterior; ERC, entorhinal cortex; PRC, perirhinal cortex.

T1-weighted images were processed using the FreeSurfer image analysis pipeline (version 5.3.0), which has been described in detail elsewhere [18], [34]. Briefly, the processing stream included removal of non-brain tissue using a hybrid watershed/surface deformation procedure [35], automated Talairach transformation, segmentation of deep gray matter (including the hippocampus and amygdala, Figure 1) using a probabilistic atlas and Bayesian classification [36], intensity normalization and topology correction [37]. Then the surface deformation was used to form tissue boundaries [38]. Once generated, the boundaries were reviewed and manually edited for technical accuracy. Then, the total gray matter volume and total intracranial volume (TIV) were calculated using FreeSurfer's automatic quantification of tissue structures. We also used the probabilistic information of tissues in T1-weighted MR images to automatically identify the white matter lesions with hypointensities [36].

Next, cerebral cortex was parcellated based on a sulcal depth-based anatomical parcellation method [39].The anatomical boundaries of ERC and PRC (Brodmann's areas 28 and 35, respectively; Figure 1) were defined according to ex vivo histology-validated MRI atlas of elderly population [25]. The parahippocampus was not measured in this study because of its partial overlap with PRC in Freesurfer labels. For ERC and PRC, we computed the average cortical thickness, surface area, cortical volume and mean curvature. The cortical thickness was calculated as the average of the distance from the white matter surface to the closest point on the pial surface and from that point back to the closest point on the white matter surface [38]. Surface area was calculated as the area of the intermediate layer between gray matter and white matter surfaces. Cortical volume at each vertex was computed by the product of the surface area and the thickness at each surface vertex. Mean curvature was the average of the two principle curvatures (1/radius of an inscribed circle) [40], and higher values represented a more steeply peaked curvature. In this study, absolute values for the mean curvature were calculated and averaged for each structure.

### Statistical analyses

Continuous variables were tested for normality using the Shapiro–Wilk test and visual inspection of histograms. Differences in demographic and clinical data were assessed using analyses of variance (ANOVA) test, and chi-square tests were used for gender.

For statistical analysis, the left and right morphometric results for each subject were combined. Analyses of covariance (ANCOVAs) were conducted to examine group differences in the morphometric results while controlling for age, gender and TIV. A two-tailed *p*<0.005 was considered significant, corresponding to a Bonferroni-adjusted significance level of *p*<0.05. Significant group effects were then further explored using Bonferroni post-hoc analyses [14]. Finally, partial correlation analyses were used for associations between brain morphometric results and cognitive scores (MMSE and MoCA) within the groups while controlling for age and gender. All statistical analyses were conducted using SPSS (version 18.0, Chicago, USA).

## Results

### Demographics

The three groups did not differ significantly with regard to age, gender or education. All the demented patients had MMSE score ≤23 and Clinical Dementia Rating (CDR) score ≥1. The MMSE and MoCA scores were significantly different across the three groups (*p*<0.001), which was driven by higher scores in the control group. There were no significant differences in MMSE and MoCA scores between the AD and BD groups. A summary of the neuropsychological test results is shown in Table 1.

### Imaging data

ANOVA tests for the volumes of the whole brain, total gray matter and WM lesions are presented in Table 2. The three groups did not differ significantly in the TIV (F[2], [41] = 0.8, *p* = 0.46). Compared with the controls, the volume of total gray matter was reduced in AD patients (*p* = 0.01), and exhibited a reduced trend in the BD group (*p* = 0.06). In addition, significantly increased volume of white matter with T1-weighted hypointensities was found in the BD patients relative to the AD patients or healthy controls (*p*<0.001).

**Table 2:** Volume (mean ± SD; in cm^3^) of brain, total gray matter and white matter with T1-weighted hypointensities.

Structure	NC (n = 16)	AD (n = 14)	BD (n = 14)	*p* value	*p* value for post-hoc analysis		
					AD - BD	NC - AD	NC - BD
TIV	1453.8±129.6	1455.0±104.6	1509.1±160.9	0.46^a^	0.87	1	0.79
Total gray matter	564.1±39.9	514.3±43.1	524.1±53.4	0.01^a^	1	0.01	0.06
WM with T1 hypointensities	2.3±1.9	4.4±2.6	24.1±1.1	<0.001^b^	< 0.001	0.96	<0.001

TIV, total intracranial volume; WM, white matter.

a *p* value for ANOVA test;

b *p* value for ANCOVA test adjusted for age, gender and TIV.

The volume of amygdala was significantly reduced in the AD and BD groups relative to the controls (*p*<0.005; Table 3). Hippocampal volume was reduced in the AD patients (*p*<0.005), and showed a reduced trend in the BD group (*p* = 0.01). The hippocampal volume in the AD group was smaller than that in the BD group, but the difference did not reach the statistical significance.

**Table 3:** Morphometric changes of medial temporal cortices.

Structure		NC (n = 16)	AD (n = 14)	BD (n = 14)	*p* value(ANCOVA)	*p* value for post-hoc analysis		
						AD - BD	NC - AD	NC - BD
HPC	Volume	4073.7±261.2	3221.1±666.9	3515.0±461.1	**<0.001**	0.21	**<0.001**	**0.01**
AMY	Volume	1565.1±166.3	1240.4±228.0	1307.3±215.0	**<0.001**	1	**<0.001**	**0.002**
ERC	Area	181.8±20.2	188.5±29.5	174.9±24.2	0.22	0.25	1	1
	Volume	1078.9±118.1	836.8±230.3	899.4±176.2	**<0.001**	0.65	**<0.001**	**0.02**
	Thickness	3.61±0.13	2.86±0.58	3.15±0.44	**<0.001**	0.14	**<0.001**	**0.03**
	Curvature	0.13±0.02	0.15±0.06	0.16±0.03	0.08	1	0.18	0.13
PRC	Area	496.8±63.3	496.5±76.2	455.4±60.0	0.05	0.09	1	0.09
	Volume	2272.8±221.7	1887.3±522.5	1946.1±380.4	**0.01**	1	**0.02**	0.07
	Thickness	3.34±0.26	2.87±0.57	3.04±0.42	**0.02**	0.51	**0.01**	0.42
	Curvature	0.14±0.01	0.17±0.03	0.17±0.03	**0.001**	1	**0.002**	**0.004**

The ANCOVA tests were adjusted for age, gender and TIV. The units of surface area, volume, thickness and mean curvature were mm^2^, mm^3^, mm and 1/mm, respectively. The numbers in bold indicate *p*<0.05, uncorrected; and those with underline represent *p*<0.005, uncorrected. AMY, amygdala; ERC, entorhinal cortex; HPC, hippocampus; PRC, perirhinal cortex.

Compared with the controls, the AD patients exhibited significantly decreased volume and cortical thickness in the ERC, and increased mean curvature in the PRC; while the BD patients only showed significant increase in mean curvature (*p*<0.005) and a reduced trend in surface area (*p* = 0.09) and volume (*p* = 0.07) in the PRC (Table 3). In addition, a strong trend for reduced volume and cortical thickness was observed for the ERC in the BD patients and the PRC in AD patients (0.005≤*p*≤0.05; Table 3). There were no significant differences in ERC and PRC measures between AD and BD patients (*p*≥0.09).

### Relationships between structural changes and cognitive decline

**Figure 2:**
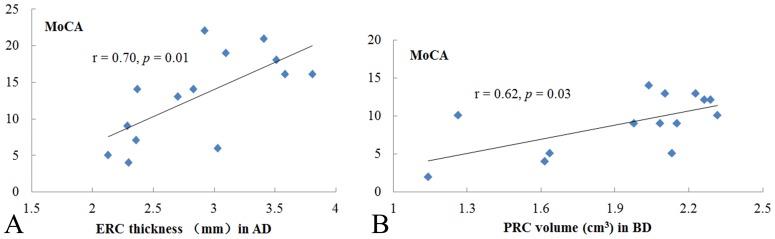
Significant correlations between MoCA scores and medial temporal measures in AD and BD patients. The correlation analyses were adjusted for age and gender.

The partial correlation analyses revealed significant associations between ERC thickness and MoCA scores (r = 0.70, *p* = 0.01) in the AD group (Figure 2A). In the BD group, only PRC volume was significantly associated with MoCA (r = 0.62, *p* = 0.03; Figure 2B) and MMSE (r = 0. 70, *p* = 0.01) scores. After adjusted for age and gender, significant correlations were also observed between the hippocampal volume and the volume of each other structure (r = 0.91, *p*<0.001 for amygdala; r = 0.93, *p*<0.001 for ERC; r = 0.95, *p*<0.001 for PRC) in the AD group. In the BD group, the hippocampal volume was also correlated with the volume of the amygdala (r = 0.60, *p* = 0.04) and ERC (r = 0.64, *p* = 0.03).

## Discussion

To the best of our knowledge, no studies have systematically investigated the morphologic changes of MTL structures in BD patients. In the current study, an automated and validated approach was used to segment the MTL cortices and the deep gray matter. Meanwhile, a wide variety of metrics was adopted to characterize geometric cortical changes in BD and AD patients. Significant volume reduction in the amygdala and shape alteration in the PRC was found in BD patients. Besides, the volume of the hippocampus, ERC and PRC also exhibited a decreasing trend. Furthermore, we found that BD and AD patients exhibited similar patterns of MTL atrophy, indicating that although atrophy of the MTL structures is a sensitive biomarker for AD, it is not sufficient for discrimination between AD and BD. Finally, structure-function correlation analyses revealed that cognitive decline was related to ERC thinning in AD and volume reduction of PRC in BD.

The volume reduction of the hippocampus and amygdala observed in BD patients confirm with earlier findings. Hippocampal lesion is common in VaD patients based upon autopsy [41], [42] and neuroimaging studies [7], [43]. The CA1 subfield of the hippocampus is known to be particularly vulnerable to excitotoxicity resulting from hypoxia and ischemia associated with vascular disease [44], and an animal study using rat model of SIVD showed substantially metabolic and histological neurodegeneration in this subfield [45]. In addition, lesion in the amygdala has also been detected in SIVD [6] or BD [8]. Considering that the amygdala belongs to the downstream organization from the hippocampal CA1 [12], and that the volume reduction of the amygdala is associated with the hippocampal atrophy observed in BD patients, we therefore speculate that the amygdala lesion may be induced by the ischemic and/or metabolic damage in the hippocampal CA1 subfield.

For BD patients, in addition to atrophy of the deep gray matter, we also found geometric alternation of the PRC in mean curvature and a reduced trend for the ERC in volume/thickness; while in AD patients, volume/thickness was significantly reduced in the ERC and showed a decreased trend in the PRC. The volume decline in AD patients is in accordance with previous manually segmented studies [16], [17], and in the ERC and PRC, the volume loss is produced primarily by the reduction in thickness, rather than in surface area [24]. Furthermore, the decreased thickness of the cerebral cortex likely represents the laminar thinning and neuronal loss in AD patients [13]. Therefore, it is suggested that AD may be partly attributed to the loss of neurons in the ERC and PRC, while PRC neurons are less affected in BD. The integrated mean curvature, on the other hand, can give a direct measure of global cortical shape in terms of how the surface is bending. One possible explanation for the increased curvature in the PRC in both AD and BD patients may be regional cortical atrophy of an inward bend, as the volume of the PRC showed a reduced trend in the two groups. Additionally, the geometric shape disturbance may be associated with the white matter atrophy, which has been reported in AD patients [46]. Further studies evaluating the possible white matter changes in BD and the exact mechanism of cortical folding are needed.

Comparisons between the AD and BD groups showed that they both exhibited similar volume reduction in the hippocampus, amygdala and ERC, as well as shape disturbance in the PRC, indicating that although atrophy of the MTL structures is a sensitive marker for AD, it is not specific. The neuroimaging data have suggested that the hippocampus and ERC were smaller in size in patients with SIVD, although not to the same extent as in AD [47]. Using a standardized visual rating scale [48], previous studies found that MTL atrophy could differentiate AD from vascular cognitive impairment [49], but not from VaD [50]. Considering that visual rating is subject to imprecise quantification and interrater variation, the current measurements were likely more accurate and comprehensive.

Our findings also provide an update on biomarkers of cognitive decline in AD and BD patients, with an emphasis on the roles of ERC thickness in AD and PRC volume in BD. These structural changes may be the core neurobiological mechanisms of the cognitive impairments in AD and BD. In AD patients, it has been shown that neurodegenerative atrophy progresses from the ERC to hippocampus, limbic system and neocortex [12], [13], [51]. Additionally, ERC thickness provides the best predictor for the conversion from MCI to AD [21] and for poorer memory performance in AD [24] than hippocampal volume or other biomarkers. Moreover, ERC thickness but not hippocampal volume was associated with cognitive decline in AD patients over one year of follow-up [18]. The ERC serves as the gateway into the hippocampal formation and receives inputs from numerous regions, including the PRC and parahippocampus [12], [52]. ERC thinning, which substantially reflects the laminar thinning and neuronal loss in this region in AD [13], may primarily contribute to the cognitive decline in AD patients.

For BD patients, the finding that PRC volume was associated with cognitive performance was surprising, as PRC volume only showed a decreasing trend in BD patients compared with controls. The PRC connects with diverse cortices, such as the cingulate cortex [53], which is an important node in the executive network [54]. It has been reported that the PRC plays an important role in recognizing novel objects [53], [55] for executive attention function and integrating item and timing information (“what” and “when”) in the service of episodic memory [56]. Therefore, the mild atrophy of the PRC in BD, rather than the white matter hyperintensity [57], may be related to the typical symptoms in BD patients, including executive dysfunction, attention impairment and progressive dementia [4]. Further investigations using a battery of specific cognitive and behavioral tests will be required to better understand the structure-function relationships.

The main limitation of the study is the relatively smaller sample size, reducing the power of comparative and correlation analyses among groups. Therefore, interpretation of these results should be done with caution. Although correction for multiple comparisons could minimize the risk of type I error, the smaller sample size would mask the subtle differences between groups or the possible relationships between imaging measures and cognitive performance. Second, although the automatic segmentation method had been widely used to examine the MTL changes, the segmentation of the MTL may be imprecise at the boundaries of subregions due to image registration and inter-subject anatomical variability. Finally, it should be noted that the subjects were not pathologically confirmed and thus misdiagnosis cannot be ruled out. Future studies may elucidate whether the structural changes in AD and BD and their associations with cognitive impairments could be detected using manual segmentation in larger sample size and autopsy-proven populations.

## Conclusion

The current study evaluated the morphologic changes of MTL cortices and deep gray matter in AD and BD patients, and determined their relationships with the cognitive impairments. There were three main findings of this study: (1) Volume reduction in the amygdala and shape disturbance in the PRC was involved in both AD and BD patients. (2) There were no significant differences in all of the structural measures between AD and BD patients, although the atrophy of most structures was more significant in AD than in BD relative to the controls. (3) Cognitive impairments were determined by ERC thickness in AD and PRC volume in BD. Overall, these results lend new insight into the impaired patterns of MTL structures in AD and BD patients, and provide an update on biomarkers for the prediction of cognition in demented patients.
